# Aryl Diazonium Chemistry for the Surface Functionalization of Glassy Biosensors

**DOI:** 10.3390/bios6010008

**Published:** 2016-03-14

**Authors:** Wei Zheng, Remko van den Hurk, Yong Cao, Rongbing Du, Xuejun Sun, Yiyu Wang, Mark T. McDermott, Stephane Evoy

**Affiliations:** 1 Department of Electrical and Computer Engineering, University of Alberta, Edmonton, Alberta, AB T6G 2V4, Canada; wzheng2@ualberta.ca (W.Z.); Remko@ualberta.ca (R.v.d.H.); 2 Department of Chemistry and National Institute for Nanotechnology, University of Alberta, Edmonton, Alberta, AB T6G 2G2, Canada; yong2@ualberta.ca (Y.C.); rdu@ualberta.ca (R.D.); mmcdermo@ualberta.ca (M.T.M.); 3 Department of Experimental Oncology, Cross Cancer Institute, University of Alberta, Edmonton, Alberta, AB T6G 1Z2, Canada; xuejun.sun@ualberta.ca; 4 Department of Chemical and Materials Engineering, University of Alberta, Edmonton, Alberta, AB T6G 1H9, Canada; yiyu@ualberta.ca

**Keywords:** biosensors, glasses, optical fibers, nanostrings, diazonium, surface linkers

## Abstract

Nanostring resonator and fiber-optics-based biosensors are of interest as they offer high sensitivity, real-time measurements and the ability to integrate with electronics. However, these devices are somewhat impaired by issues related to surface modification. Both nanostring resonators and photonic sensors employ glassy materials, which are incompatible with electrochemistry. A surface chemistry approach providing strong and stable adhesion to glassy surfaces is thus required. In this work, a diazonium salt induced aryl film grafting process is employed to modify a novel SiCN glassy material. Sandwich rabbit IgG binding assays are performed on the diazonium treated SiCN surfaces. Fluorescently labelled anti-rabbit IgG and anti-rabbit IgG conjugated gold nanoparticles were used as markers to demonstrate the absorption of anti-rabbit IgG and therefore verify the successful grafting of the aryl film. The results of the experiments support the effectiveness of diazonium chemistry for the surface functionalization of SiCN surfaces. This method is applicable to other types of glassy materials and potentially can be expanded to various nanomechanical and optical biosensors.

## 1. Introduction

Biological detection is of importance in many fields such as disease biomarker diagnosis and monitoring, drug discovery, and molecular identification [1,2,3,4,5,6,7]. For instance, the multiplexed assaying of proteins enables the understanding and diagnosis of complex diseases where traditional genomic assays are somewhat inadequate. Prions play a significant role in diseases, such as the Creutzfeldt-Jakob disease and transmissible spongiform encephalopathies [8,9]. In turn, peptides, such as the amyloid-beta (Aβ), are recognized causative agent of degenerative conditions such as Alzheimer’s disease [9,10,11]. Protein microarrays enable multiplexed assays and have been employed to quantify cancer cells and biomarkers [12,13]. In addition, protein misfolding cyclic amplification is used for the testing of prions and the diagnosis of related diseases [14,15,16,17]. The technique however involves multiple steps and is inappropriate for large scale screening. 

Label-free biosensors have been looked upon as alternative platforms for such assays [18,19]. Such biosensors typically integrate a bio-recognition probe with a transduction system, as well as electronic systems such as signal amplifiers, processors, and display. Transduction platforms, such as quartz crystal microbalance (QCM) [20,21,22,23], micromechanical resonators [24,25,26,27,28,29,30], flow cytometry [18,31,32,33], amperometry [34,35,36,37,38], surface plasmon resonance (SPR) [39,40,41,42,43,44,45,46], and planar optical waveguides [47,48,49], have been investigated. In turn, numerous biological probes such as DNA [50], RNA [51], monoclonal [52,53] polyclonal antibodies [54], bacteriophages [35,55,56,57,58,59] and their binding proteins [58,60] have been leveraged to impart specificity to the transduction. Most of these sensing platforms involve the immobilization of the probe/target system onto a metallic or semiconducting surface. The last few years have however seen the emergence of alternate transduction mechanisms that rather involve the interaction of the analyte with an optical field propagating in glassy waveguides [61,62,63,64,65,66,67,68,69,70]. More specifically, fiber-optic sensors offer the advantages of miniaturization, multiple detection, low loss remote monitoring, and large information contents [69]. Further, advancement in the nanomachining of glasses [71,72] has enabled the realization glassy sensing nanostrings [73,74] as narrow as 8 nm [75].

The use of stable interfacial moieties offering strong adhesion, biocompatibility, protection against corrosion and long term stability is crucial to the realization of reliable biosensors [76,77,78]. Chemical bonding is usually preferred over simple physisorption as it provides better coverage and more stable linkage. Electrochemistry is commonly employed to insure optimal coverage and reliability. Electrochemical approaches are however only suitable for conductive and semi-conductive materials and are thus not applicable to the functionalization of insulating glasses. Electrochemistry also requires complex apparatus, can involve harsh chemicals and lead to the deterioration of the biological system [79,80]. Hence, a simpler, milder, versatile and biocompatible process for the linking of biomolecules onto glasses would be required.

A recently reported diazonium salt reduction induced aryl film grafting process represents a potent candidate for such applications [79,80]. This diazonium chemistry involves a single salt redox process, is implemented in aqueous solution at room temperature and does not require complicated equipment. The mild conditions involved insure process compatibility with pre-existing biological layers. Furthermore, diaznonium chemistry forms covalent bonding onto the surface, thus offers high thermal, mechanical and ambient stability. Moreover, this chemical modification is applicable to all types of materials, from metals to insulator, organic to inorganic. 

**Figure 1:**
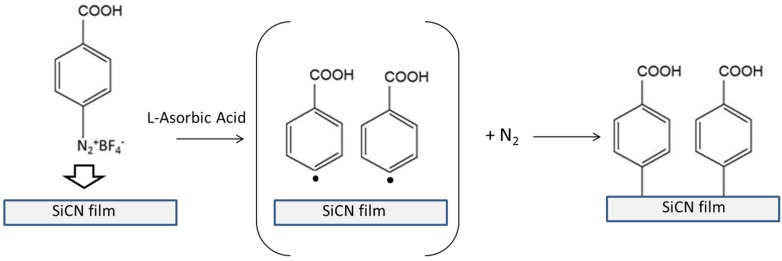
Diagram of diazonium aryl functionalization of SiCN surfaces.

We here report a diazonium salt induced aryl film grafting process to modify a novel SiCN glassy material [28,72,81,82] (Figure 1). Various surfaces, ranging from metal [83], semi-conductor [84] carbon [85,86] and non-conductive materials [79,80] can be modified by the reduction of aryldiazonium salts by either electrochemically or chemically. As shown in Figure 1, the surface modification is based on radical reactions. Reduction of diazonium salts generates reactive aryl radicals, which then bind to SiCN surfaces. This attachment of the aryl film on substrate surface is very stable [83]. The covalent interface bonding on carbon [87,88,89], graphene [90,91], silicon [92,93], copper [94], and gold [95,96,97] surfaces has thus been reported.

**Figure 2:**
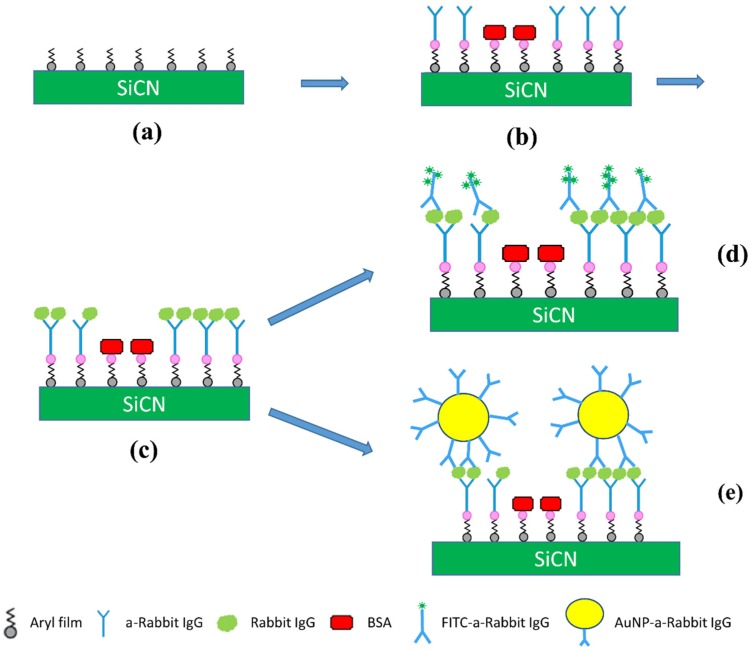
Schematic diagram of rabbit IgG sandwich assay onto SiCN surfaces. (**a**) grafting of aryl groups, as described in Figure 1; (**b**) activation of carboxyl groups and attachment of anti-rabbit IgG and BSA blocking layer; (**c**) specific capture of rabbit IgG; (**d**) tagging using FITC-labelled anti-rabbit IgG; (**e**) tagging using gold nanoparticles functionalized with anti-rabbit IgG.

In a prior report, we employed such grafting process for the functionalization of resonant SiCN nanostrings [98]. In that work, the grafting was employed to covalently attach anti-rabbit IgG as a molecular probe. Specific enumeration of rabbit IgG was successfully performed through observation of downshifts of the resonant frequencies. In the work reported here, a similar grafting process is rather used to enable a sandwhich assay. More specifically, rabbit IgG binding assays are performed on the diazonium treated SiCN surface. Fluorescently-labelled rabbit IgG and gold nanoparticles (AuNPs) conjugated with rabbit IgG were used individually as markers to demonstrate the absorption of anti-rabbit IgG and further verify the successful use of aryl films for the functionalization of SiCN surfaces (Figure 2).

## 2. Results and Discussion

### 2.1. SiCN Film Composition

**Figure 3:**
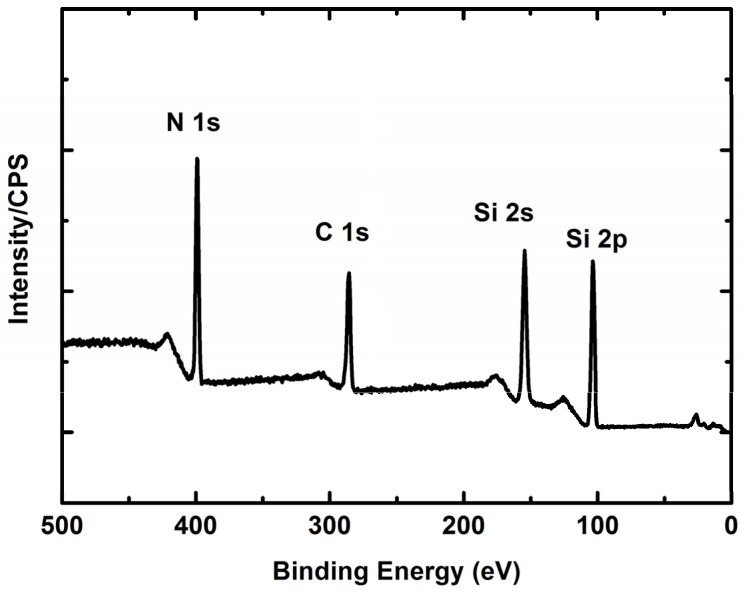
XPS survey of SiCN film.

Figure 3 displays an X-ray photoelectron spectroscopy (XPS) survey of a typical SiCN film. Significant binding energy peaks are observed for silicon, carbon and nitrogen. Data analysis yields the quantitative elemental composition of the films (Table 1). The atomic Si:C:N composition ratio was roughly 4:3:3.

### 2.2. SEM and EDX of AuNPs

**Figure 4:**
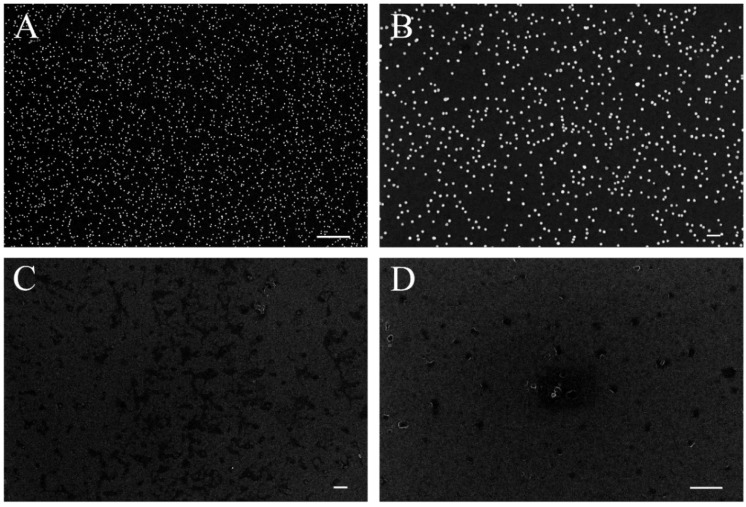
Comparison of presence of AuNP on the surface of sample chip A1 and negative control chip C1: (**A**) AuNP distribution on chip A1 under 10,000x magnification, scale bar length is 1 μm; (**B**) AuNP distribution on chip A1 under 20,000x magnification, scale bar length is 200 nm; (**C**) AuNP distribution on chip C1 under 10,000x magnification, scale bar length is 1 μm; (**D**) AuNP distribution on chip C1 under 20,000x magnification, scale bar length is 200 nm.

**Figure 5:**
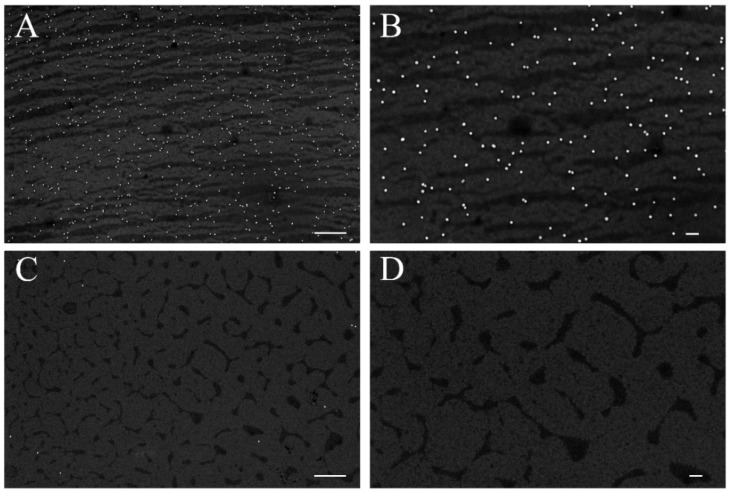
Comparison of presence of AuNPs on the surface of sample chip B1 and negative control chip D1: (**A**) AuNP distribution on chip B1 under 10,000 times magnification, scale bar length 1 μm; (**B**) AuNP distribution on chip B1 under 20,000 times magnification, scale bar length 200 nm; (**C**) AuNP distribution on chip D1 under 10,000 times magnification, scale bar length is 1 μm; (**D**) AuNP distribution on chip D1 under 20,000 times magnification, scale bar length is 200 nm.

Figure 4 shows scanning electron microscope (SEM) images of AuNPs on sample A1 (Figure 3B and Figure 4A) and control chip C1 (Figure 4C,D). The images show a dense and roughly uniform distribution of AuNPs over the surface. Almost no AuNP were observed on the control chip C1. It is noteworthy that these results were obtained using high concentrations of anti-rabbit IgG-conjugated AuNPs. To exclude the possibility of non-specific binding by saturation, a 1:3 dilution of anti-rabbit IgG conjugated AuNPs solution was applied to sample chip B1 and control chip D1. The results are shown in Figure 4. Sample B1 shows a roughly uniform coverage of AuNP but at a lower distribution density compared to that of A1. Very small amounts of AuNP are present on control chip D1, again pointing to a negligible level of non-specific binding to the surface. This minimal amount of non-specific binding is likely caused by the interaction between the polyclonal goat anti-rabbit IgG and the goat IgG. The interaction of the AuNPs themselves to the IgG molecules on the substrate may however be caused by other factors. No AuNP are shown under magnification of 20,000x. The contrast of AuNP density between samples and negative controls observed in both experiments (*i.e.*, Figure 4 and Figure 5) therefore points to a high selectivity of binding of anti-rabbit IgG conjugated AuNPs onto the surface.

**Figure 6:**
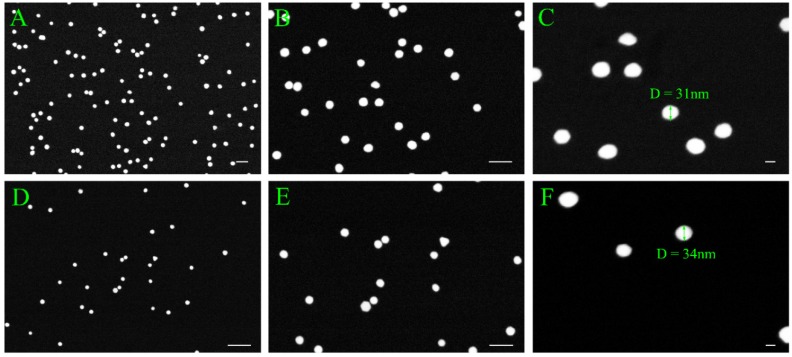
Comparison of presence and density of AuNP on the surface of chips A1 and B1 under high magnification: (**A**) AuNP distribution on chip A1 under 50,000 times magnification, scale bar stands for 100 nm; (**B**) AuNP distribution on chip A1 under 100,000 times magnification, scale bar stands for 100 nm; (**C**) AuNP distribution on chip A1 under 200,000 times magnification, scale bar stands for 20 nm; (**D**) AuNP distribution on chip B1 under 50,000 times magnification, scale bar stands for 200 nm; (**E**) AuNP distribution on chip B1 under 100,000 times magnification, scale bar stands for 100 nm; (**F**) AuNP distribution on chip B1 under 200,000 times magnification, scale bar stands for 20 nm.

**Figure 7:**
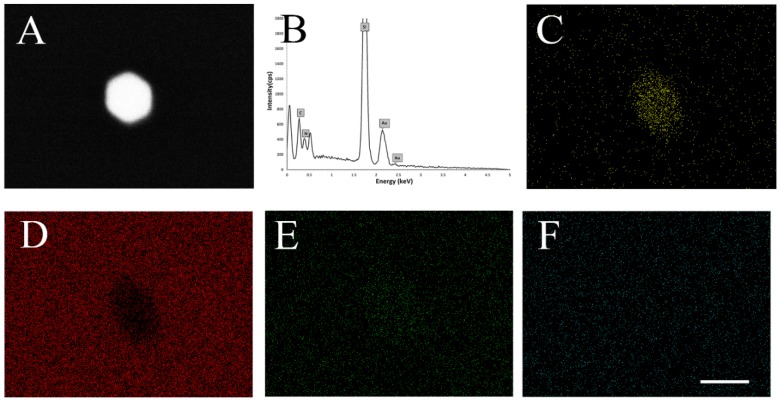
EDX analysis of substrate immobilized with AuNP: (**A**) SEM image of AuNP; (**B**) EDX spectrum of a point of the particle, showing peaks of gold, silicon, carbon and nitrogen; (**C**) EDX map of distribution and relative intensity of gold in scanned area; (**D**) EDX map of distribution and relative intensity of silicon in scanned area; (**E**) EDX map of distribution and relative intensity of carbon in scanned area; (**F**) EDX map of distribution and relative intensity of element nitrogen in scanned area. Scale bar stands for 50 nm.

Figure 6 compares the density of AuNPs on chips A1 and B1. Under 50,000x and 200,000x magnification, the ratio of AuNP of A1 to B1 is around 3:1 which is consistent with the relative concentrations of anti-rabbit IgG conjugated AuNP solutions concentration employed in the two experiments. Figure 7 shows the EDX analysis of SiCN substrate immobilized with sandwich rabbit IgG structure. The point analysis of the particle (panel B) shows significant peaks for elements gold, silicon, carbon and nitrogen, which further supports the attachment of AuNPs onto the SiCN substrate. The elements map shows their relative proportions on and off the particle. In spite of background noise of sparse gold distribution of the particle, the contrast of gold density on and off particle is high enough to conclusively establish the presence of discrete AuNPs on the surface (panel C). Panels D, E, F show less silicon, a bit more carbon and almost the same amount of nitrogen on the particle, which agrees with the fact that anti-rabbit IgG conjugated AuNPs contains carbon and nitrogen, but no silicon. The shape of elements mapping are slightly distorted due to the electron astigmation at high magnification but they still reflects the concentrations of elements distribution effectively.

### 2.3. Fluorescence Imaging

**Figure 8:**
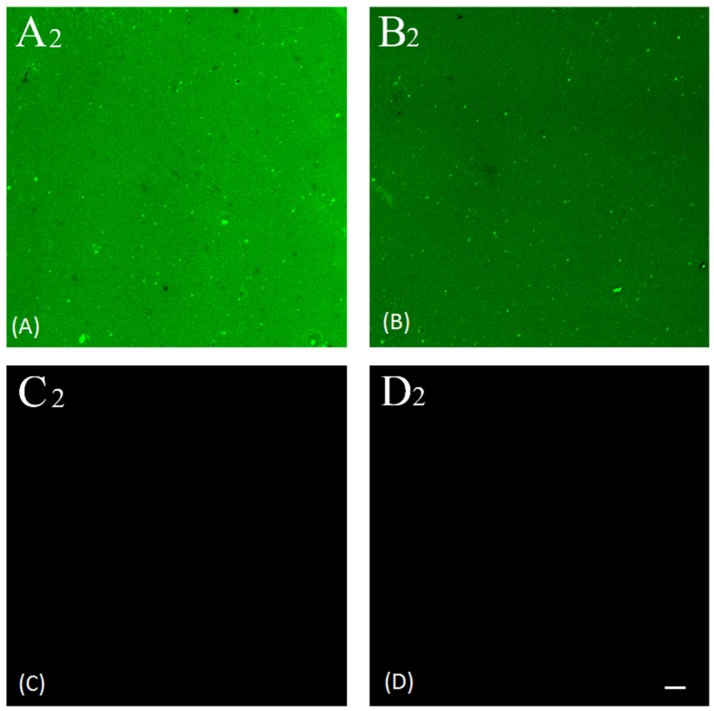
Comparison of presence of FITC on the surface of sample chip A2, B2 and control chip C2, D2: (**A**) FITC distribution on chip A2; (**B**) FITC distribution on chip B2; (**C**) FITC distribution on chip C2; (**D**) FITC distribution on chip D2. Scale bar stands for 2 μm.

As seen in Figure 8, the fluorescein-5-Isothiocyanate (FITC) fluorescent marker was present only in chips A2 and B2 but not in negative control chips C2 and D2. The FITC fluorescent intensity is clearly stronger in A2 than it is in B2, while almost no difference exists between C2 and D2. Table 2 tabulates the mean fluorescent intensity and signal to noise ratio for those samples. The signal to noise ratios of 185 and 105 under FITC dilution of 1:200 and 1:400, respectively, conclusively establishes the selective absorption of FITC-conjugated anti-rabbit IgG to the target molecule rabbit IgG. The fluorescence intensity of A2 is 47% larger than that of B2, suggesting non-saturation binding of FITC conjugated anti-rabbit IgG to substrate. The intensities from negative control samples C2 and D2 are almost the same for both FITC concentration, further pointing to an almost inexistent binding of FITC conjugated anti-rabbit IgG to substrate. Hence, these results conclusively establish that the chemical bonding of probe molecule anti-rabbit IgG to the aryl film on SiCN substrate induced by diazonium modification has been accomplished.

## 3. Materials and Methods

### 3.1. SiCN Film Deposition

A previously reported deposition technique was employed to deposit the thin film [28,72]. More specifically, a single-crystal (100) silicon wafer was subjected to a 15 min piranha clean (3:1 H_2_SO_4_:H_2_O_2_) to eliminate surface organics, and immediately followed by a 3 min buffered oxide etch (BOE, 10:1 HF:NH_4_F) to strip the native oxide. The SiCN film was deposited onto the clean silicon wafer by plasma enhanced chemical vapor deposition (PECVD). First, the PECVD chamber (Trion Orion) was pre-conditioned at a mixed gases flow of 25 sccm DES, 40 sccm NH_3_, 55 sccm N_2_ at a temperature of 300 °C, a pressure of 500 mTorr, and a power of 50 W for 1200 s. The silicon wafer was then loaded in the reactor. The chamber was purged by 55 sccm N_2_ at a pressure of 500 mTorr and a temperature of 300 °C for 10 s. The chamber was prepared for the deposition by a mixed gases flow of 25 sccm DES, 40 sccm NH_3_, 55 sccm N_2_ at a temperature of 300 °C, a pressure of 500 mTorr for 10 s. A SiCN thin film was deposited with at a mixed gases flow of 25 sccm DES, 40 sccm NH3, 55 sccm N2 at a temperature of 300 °C, a pressure of 500 mTorr, and a power of 50 W for 125 s. The wafer was then annealed in a 3-zone Minibrute tube furnace at 525 °C for 2 h.

### 3.2. XPS Element Analysis

Surface XPS analyses were performed to characterize the elemental composition of the deposited film, namely the ratio of silicon, carbon and nitride. The XPS was performed in a Kratos Axis Ultra spectrometer using a monochromatic Al Kα source (hν = 1486.6 eV). Instrument base pressure was lower than 5 × 10^−8^ Pa. The survey scans were acquired out with a pass energy of 160 eV.

### 3.3. Diazonium Modification of SiCN

Aryl diazonium salt was synthesized from the corresponding anilines using a previously reported procedure [86]. Briefly, 0.1 moles 4-Aminobenzoic acid (Sigma-Aldrich) was dissolved in 50 mL floroboric acid (48%, Sigma-Aldrich) and then cooled in an ice water bath. After cooling the solution to 0 °C, Sodium nitrite (10 g, Sigma-Aldrich) dissolved in DI water (20 mL) was added drop by drop with stirring. The reaction mixture was further cooled in ice water bath and stirred for another 1 h. The resultant precipitate was filtered in a Buchner funnel and washed with cold anhydrous ether (Sigma-Aldrich). 

The SiCN surface was modified by diazonium salt reduction induced aryl film grafting process as per the following procedure. A piranha and BOE etch similar to the one described previously was first employed to clean the SiCN surface from organic contaminants. Indeed, the SiCN films were batch deposited on multiple wafers at a time, with individual wafers often stored for months prior to use. A re-cleaning of the SiCN film surface prior to aryl grafting was thus deemed compulsory in the light of this extensive storage. Following this cleaning, the SiCN chips were incubated in 2:1 volume ratio of 0.05 M 4-carboxybenzenediazonium tetrafluoroborate (COOH−C_6_H_4_−N_2_BF_4_) and 0.05 M L-ascorbic acid (VC) (Sigma-Aldrich) for 60 min. The chips were then successively rinsed in water, ethanol, and acetone followed by a 5 min sonication in dimethylformamide (DMF) (Sigma-Aldrich). The presence of grafted aryl film onto the SiCN film after sonication in DMF was verified with XPS analysis. For such verification, a Br terminated diazonium salt 4-bromobenzenediazonium tetrafluoroborate was rather used. Bromium was used as elemental marker as it is otherwise absent from the SiCN film surface. Related XPS spectra clearly indicated the presence of the Br groups following the sonication step in DMF, confirming the stability of the grafting [98].

Carboxyl groups were activated by incubating the chips in a 1:1 volume ratio of 0.4 M EDC (N-(3-dimethylaminopropyl)-N′-ethylcarbodiimide hydrochloride) (Sigma-Aldrich) and 0.1 M NHS (N-hydroxysuccinimide) (Sigma-Aldrich) solution for 30 min.

### 3.4. Gold Nanoparticle Labelled Sandwich Rabbit IgG Assay

In this AuNP labelled sandwich rabbit IgG assay, anti-rabbit IgG was used as the recognition bioreceptor, rabbit IgG was the target and anti-rabbit IgG conjugated AuNP served as detection markers.

#### 3.4.1. Immobilization of AuNP Labelled Sandwich Rabbit IgG

*Immobilization of recognition bioreceptor*: Four SiCN chips (A1, B1, C1, D1) baring activated carboxyl groups were incubated in 100 µg/mL goat anti-rabbit IgG solution (polyclonal, Sigma-Aldrich) at room temperature for 2 h. The chips were rinsed with PBST (Phosphate Buffered Saline Tween-20) and then incubated in 5% BSA (‎bovine serum albumin) at room temperature for 1 h to block non-specific binding sites. The chips were then rinsed once again in PBST.

*Immobilization of target:* Two chips (A1 and B1) were immobilized with the detection target by incubating in 200 µg/mL rabbit IgG solution (polyclonal, Sigma-Aldrich) at room temperature for 1 h. As parallel control experimnent, the other two chips (C1 and D1) were incubated in 200 µg/mL goat IgG solution (polyclonal, Sigma-Aldrich) at room temperature for 1 h. The chips were then rinsed once again in PBST.

*Immobilization of detection marker:* Chips A1 and C1 were immersed in 40 nm AuNP (4.5 × 10^11^ /mL) conjugated anti-rabbit IgG solution (10 µg/mL, Ted Pella) at room temperature for 1 h. Chips B1 and D1 were immersed in 1:3 diluted solution of 40 nm AuNP conjugated anti-rabbit IgG at room temperature for 1 h. Following incubation, the chips were water rinsed and dried under nitrogen flow. Table 3 summarizes the conditions under which each sample was prepared.

#### 3.4.2. SEM and EDX

A high resolution field emission scanning electron microscopy (Zeiss Sigma FE-SEM) was used to observe the presence of the gold nanoparticles on the surface of chips A1, B1, C1 and D1. Images were obtained at magnifications ranging from 10,000x to 200,000x using an acceleration voltage of 15 KV, and using a secondary electron (SE) in-lens and backscattered electron (BSE) detector. An energy-dispersive X-ray detection instrument (EDX) was used to identify the elemental composition of surfaces of chips A1, B1, C1 and D1. An Oxford Instruments X-Max^N^ 150 mm^2^ Silicon Drift Detector (SDD) detector was used for such point analysis and mapping of elemental spatial distributions.

### 3.5. FITC Labelled Sandwich Rabbit IgG Assay

The FITC labelled sandwich rabbit IgG assay was similar to the AuNP labelled sandwich rabbit IgG assay with the exception that FITC conjugated anti-rabbit IgG was used as the detection marker.

#### 3.5.1. Immobilization of FITC Labelled Sandwich Rabbit IgG 

The procedure for immobilization of bioreceptor and target is similar to the one described in section 4.4.1. Four diazonium modified SiCN chips (A2, B2, C2, D2) were incubated in 100 µg/mL goat anti-rabbit IgG solution (polyclonal, Sigma-Aldrich) for 2 h, and 5% BSA (‎bovine serum albumin) for 1 h at room temperature. Chips A2 and B2 were incubated in 200 µg/mL rabbit IgG solution (polyclonal, Sigma-Aldrich) for 1 h whereas chips C2 and D2 were subject to 200 µg/mL goat IgG solution (polyclonal, Sigma-Aldrich) as control for 1 h at room temperature. Chips A2 and C2 were immersed in 1:200 diluted FITC conjugated anti-rabbit IgG solution (Sigma-Aldrich) while Chips B2 and D2 were immersed in 1:400 diluted FITC conjugated anti-rabbit IgG solution at room temperature for 1 h.

#### 3.5.2. Confocal Microscopy Imaging

Chips A2, B2, C2, D2 were individually imaged on a Zeiss LSM 710 Laser scanning confocal microscope mounted on an Axio-observer inverted microscope (ZEN 2011, Jena, Germany) with a plan Apochromat 20x (NA 0.8) dry lens. Fluorescence signal was collected with a 488 nm laser and with an emission wavelength ranging from 492 nm to 590 nm. Images were digitized at 16 bit with a Nyquist sampling rate using a pinhole size of one airy unit. The average intensity of individual images were calculated using the imageJ software (National Institutes of Health).

## Figures and Tables

**Table 1:** Element composition analysis of SiCN film.

Element	Atomic Concentration %	Mass Concentration %
N 1s	29.69	21.94
C 1s	30.87	19.57
Si 2p	39.46	58.49

**Table 2:** Mean optical intensity of FITC on the surface of sample chip A2, B2 and control chip C2, D2.

Sample	Intensity (AU)	Signal to Noise Ratio	Dilution Ratio of FITC Conjugated Anti-Rabbit IgG
A2	37,967	185	1:200
C2	205		
B2	25,791	105	1:400
D2	245		

**Table 3:** Description of Samples.

Sample	Control Type	Dilution Ratio	Label
A1	positive	1:1	AuNP
B1	positive	1:3	AuNP
C1	negative	1:1	AuNP
D1	negative	1:3	AuNP
A2	positive	1:200	FITC
B2	positive	1:400	FITC
C2	negative	1:200	FITC
D2	negative	1:400	FITC

## Abbreviations

The following abbreviations are used in this manuscript:

NEMS	Nano-electromechanical Systems
AuNP	Gold nanoparticle
SEM	Scanning electron microscopy
XPS	X-ray photoelectron microscopy
PBST	Phosphate Buffered Saline Tween-20
PECVD	Plasma enhanced chemical vapor deposition
FITC	Fluorescein-5-Isothiocyanate
